# A case of recurrence of adult-onset Still’s disease in the third trimester: a case report and literature review

**DOI:** 10.1186/s12884-021-03648-1

**Published:** 2021-02-24

**Authors:** Satoshi Hosoya, Miyuki Sadatsuki, Shinji Izuka, Hiroyuki Yamashita, Hajime Oishi

**Affiliations:** 1 Department of Obstetrics and Gynecology, National Center for Global Health and Medicine, 1-21-1, Toyama, Shinjuku-ku, Tokyo, 162-8655 Japan; 2 Division of Rheumatic Diseases, National Center for Global Health and Medicine, Tokyo, Japan

**Keywords:** Adult-onset Still’s disease, HELLP syndrome, IL-18, Third trimester, Pregnancy

## Abstract

**Background:**

Adult-onset Still’s disease (AOSD) is a self-inflammatory disease showing macrophage and neutrophil activation by inflammatory cytokines such as TNF-α, IL-6, and IL-18. Although some cases with the flare of AOSD during pregnancy have been reported, most had flares in the first or second trimester and few had flares in the third trimester. In this report, we present the case of a patient with recurrent flare of AOSD in the third trimester and discuss the management of AOSD in the third trimester with the review of previous literatures.

**Case presentation:**

A 38-year-old woman in complete AOSD remission without medication presented with impaired liver function, low platelet count, mild fever, abdominal pain, splenomegaly, and elevated ferritin and IL-18 levels at 30 gestational weeks. Although the laboratory data and physical examination finding suggested HELLP syndrome or acute fatty liver of pregnancy and we considered the termination of her pregnancy, her fetus was in a reactive status. Considering her fetal status, some specific findings of AOSD, and her AOSD history, we and rheumatologists diagnosed her with AOSD recurrence and started systemic steroid therapy. In her clinical course, three flares of AOSD occurred, twice in the third trimester and once in postpartum; twice systemic steroid pulse therapies were then needed. Ultimately, a healthy infant was delivered transvaginally at 36 gestational weeks spontaneously.

**Conclusions:**

Specific findings of the flare of AOSD such as fever, splenomegaly, elevated ferritin and IL-18 levels, and fetal status could be useful findings for differentiation from HELLP syndrome and AFLP in the third trimester. With the careful management supported by rheumatologists, patients complicated with the flare of AOSD may continue their pregnancy longer than we expected.

## Background

Adult-onset Still’s disease (AOSD) is a self-inflammatory disease exhibiting macrophage and neutrophil activation by inflammatory cytokines such as tumor necrosis factor-α (TNF-α), interleukin-6 (IL-6), and interleukin-18 (IL-18). Its incidence is 1.6–4.0 in 1,000,000 people per year [1]. AOSD more likely occurs in women, affecting 50 to 70%, than in men [1]. It mostly affects young adults (median age at diagnosis: 36 years) [1], indicating the reproductive ages in women. The Yamaguchi criteria [2] are commonly used to diagnose AOSD because they suggest major symptoms and laboratory findings of AOSD; common manifestations are remittent fever over 39 °C lasting more than a week, arthralgia or arthritis, typical rash, high white blood cell count, elevated liver enzymes, and splenomegaly (Table 1). In addition, some AOSD cases manifest abdominal pain and nausea [1]. If findings such as elevated liver enzyme and abdominal pain happen during pregnancy, AOSD may be misinterpreted as hemolysis, elevated liver enzymes and low platelet count (HELLP) syndrome and acute fatty liver of pregnancy (AFLP). Currently, although the association between the disease onset of AOSD and pregnancy has already been widely investigated [3, 4], such relationship remains unclear. Moreover, reported cases of AOSD flares at third trimester are few, and the management and features of AOSD, which has similar features to HELLP syndrome and AFLP, at the third trimester have not been reported yet. Herein, we report a rare case of a pregnant woman with AOSD recurrence at her 30 gestational weeks and reviewed the previous reports on the flares of AOSD related to pregnancy. Written informed consent for the publication of this report was obtained from the patient.

**Table 1:** Yamaguchi criteria for the diagnosis of adult-onset Still’s disease [2]

Major criteria	Fever ≥39 °C lasting ≥1 week
	Arthralgia or arthritis lasting ≥2 weeks
	Typical nonpruritic salmon-colored rash
	Leukocytosis ≥10,000/μl with granulocytes ≥80%
Minor criteria	Sore throat
	Lymphadenopathy
	Splenomegaly
	Abnormal liver function tests
	Negative tests for antinuclear antibody and rheumatoid factor
Exclusion criteria	Infection
	Malignancy
	Other rheumatic disease (vasculitis)

Diagnosis of adult-onset Still’s disease if ≥5 criteria are present with ≥2 being major criteria and no exclusion criteria

## Case presentation

**Fig. 1:**
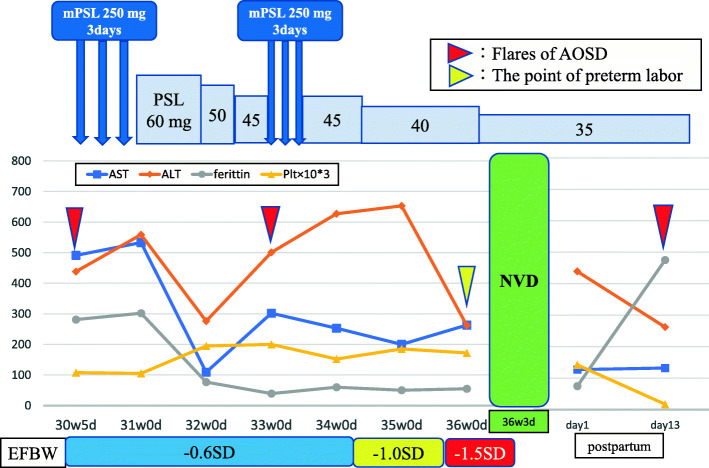
Clinical course, laboratory data, and treatment of the patient. Initial mPSL pulse therapy was provided for 3 days after 30 weeks and 5 days of gestation and then switched to oral PSL therapy. At 33 weeks and 0 days of gestation, another pulse therapy was provided for the second flare. EFBW was restricted gradually from − 0.6 SD to − 1.5 SD. At 36 weeks and 0 days of gestation, this patient started irregular uterine contractions and finally delivered a healthy infant spontaneously at 36 weeks and 3 days of gestation. At 12 days postpartum, she experienced the third flare, and oral PSL therapy was maintained

A 38-year-old primigravida with well-controlled gestational diabetes mellitus (GDM) was introduced from her home doctor to our hospital, presenting with mild fever, liver dysfunction, elevated ferritin, low platelet count, and proteinuria at 30 weeks and 5 days of gestation. She was diagnosed with AOSD 6 years ago in our hospital based on typical symptoms, including fever, malaise, cervical lymphadenopathy, rash, liver disfunction, and high serum ferritin levels. Although the rheumatologists performed biopsies of her bone marrow and lymph node, the results of these examination were normal and the possibility of other hematologic diseases was denied. After the combination treatment with systemic steroid and tocilizumab administration, she was in complete clinical remission without medication at conception. Upon arrival, her body temperature, blood pressure, and heart rate were 37.7 °C, 101/63 mmHg, and 103 beats/min. In systemic physical examinations, no abnormalities were found. The laboratory findings at arrival are presented in Table 2; The results indicated liver dysfunction, low platelet, and elevated IL-18 and aldolase levels. No apparent coagulopathy findings were observed. Basic infectious disease tests including Epstein–Barr virus and cytomegalovirus were all negative. Nonstress test and fetal sonography revealed perfect biophysical profile scoring of her fetus, mild polyhydramnios (maximum vertical pocket of amnion: 8.3 cm), and an estimated fetal body weight (EFBW) of 1663 g (+ 0.8 standard deviation [SD]). However, splenomegaly was detected by additional abdominal sonography. Although HELLP syndrome or AFLP was suspected upon admission according to liver dysfunction and low platelet count at third trimester and termination of pregnancy could be possible, her clinical course and examination results did not match well to Sibai’s criteria for HELLP syndrome [5] and Swansea’s criteria for AFLP [6]. Moreover, her general condition and fetal status did not require urgent intervention. Considering the findings such as mild fever, elevated ferritin level, high level of IL-18, splenomegaly, and good fetal status, she was ultimately diagnosed with recurrence flare of AOSD by us and some rheumatologists. Thus, systemic steroid pulse therapy (first dose: 250 mg of methylprednisolone [mPSL] for 3 days) was initiated and switched to oral prednisolone (PSL) thereafter. Additionally, the patient was carefully followed up by frequent laboratory tests and transabdominal fetal sonography. Figure 1 illustrates her clinical course after hospitalization at 30 gestational weeks. Clinical AOSD findings such as fever, liver dysfunction, and high ferritin level were well controlled for 3 weeks after the initiation of systemic steroid therapy; hence, the oral PSL dosage was gradually reduced. However, another steroid pulse therapy was needed for the second flare of AOSD that happened at her 33 gestational weeks with elevated liver enzymes and rash on her trunk. In her clinical course, although with maintained uneventful fetal status, the growth of the fetus had been gradually restricted, resulting in intrauterine growth restriction (IUGR). At 36 weeks and 0 days of gestation, she had irregular uterine contractions without apparent infectious findings, and preterm premature rupture of membrane (pPROM). Finally, she spontaneously delivered a female infant transvaginally at 36 weeks and 3 days of gestation. Her infant had a low birth weight (2440 g) but was very healthy; the Apgar score was 9/10, and pH of the umbilical artery was 7.312. Moreover, no abnormal findings of the amniotic fluid at delivery and no pathological features in her placenta and umbilical cord were found. At 12 days postpartum, she had elevated ferritin levels and extremely low platelet count, indicating the third AOSD flare by a rheumatologist; thus, the dosage of oral PSL was increased and maintained for months. During her follow-up checkup as an outpatient by rheumatologists at 1 year postpartum, her C-reactive protein (CRP) and ferritin levels were within the reference range, and her platelet count gradually increased, indicating a well-controlled AOSD.

**Table 2:** Laboratory findings

	Value	Reference intervals, unit
T-Bil	0.7	0.4–1.5, mg/dl
AST	491	13–30, U/L
ALT	438	7–23, U/L
LDH	653	124–222, U/L
γ-GTP	10	9–32, U/L
BUN	6.4	8.0–20.0, mg/dl
Cre	0.58	0.46–0.79, mg/dl
BS	84	80–110, mg/dl
UA	3.3	2.6–7.0, mg/dl
CRP	1.81	0.00–0.14, mg/dl
TG	163	30–149, mg/dl
Ferritin	281	5–157, ng/ml
WBC	2980	3300–8600, /μl
RBC	3.93	3.86–4.92, × 10^6^/μl
Hb	10.7	11.6–14.8, g/dl
Ht	32.5	35.1–44.4, %
Plt	108	158–348, × 10^3^ /μl
PT-INR	0.89	0.90–1.10
APTT	33	25–35, seconds
Fib	407	200–400, mg/dl
AT III	96	80–130, %
Urinary protein	0.33	0.02–0.12, g/day
IL-18	175,116	0–211, pg/ml
Aldolase	30.6	2.5–7.5, U/L

## Discussion and conclusions

The flare of AOSD in pregnancy was first reported in 1980 by Stein et al. [7]. Currently, nearly 30 reports about AOSD associated with pregnancy have been published [3, 4, 7–32]. Hence, we reviewed the cases of AOSD complications during pregnancy [3, 4, 7–32]. A total of 53 flares in 47 pregnant women, including our case, have been recorded. The clinical course of AOSD during pregnancy consists of three types: first-onset type, which refers to the first onset of AOSD related to pregnancy [3, 4, 7, 8, 11, 12, 14, 17–25, 28–30, 32], recurrent-flare type, which refers to a recurrent flare related to pregnancy with previous AOSD diagnosis [4, 7, 9, 13, 14, 16, 25, 31], and no-flare type, which is only a complication with no flare related to pregnancy [4, 9, 10, 14, 15, 17, 26, 27]. In the literature review, the cases were divided into these three types shown in Table 3 and our case belongs to the recurrent-flare type.

**Table 3:** Review of pregnancy cases with adult onset Still’s disease classified into three types

Total: 47 pregnant women and 53 pregnancies			
	First onset (Number = 26)	Recurrent flare (Number = 13)	No flare (Number = 14)
Age at pregnancy (year), Median [IQR]	29 [25–33] (1 unknown case)	31 [29–34]	27 [26–30] (1 unknown case)
Parity, %			
Primigravida	50 (8/16)	80 (8/10)	69.2 (9/13)
Multigravida	50 (8/16)	20 (2/10)	30.8 (4/13)
Unknown, number	10	3	1
Timing of flare, %			
1st trimester	24 (6/25)	23.1 (3/13)	–
2nd trimester	52 (13/25)	38.5 (5/13)	–
3rd trimester	8 (2/25)	7.7 (1/13)	–
Postpartum	16 (4/25)	30.8 (4/13)	–
Unknown, number	1	0	–
Major symptoms and complications, %			
Fever	95.7 (22/23)	70 (7/10)	–
Arthralgia, Arthritis	91.3 (21/23)	60 (6/10)	–
Erythema, Rash	78.3 (18/23)	50 (5/10)	–
Sore throat	65.2 (15/23)	30 (3/10)	–
Lymphadenopathy	60.9 (14/23)	10 (1/10)	–
Hepatomegaly, Liver dysfunction	34.8 (8/23)	30 (3/10)	–
Splenomegaly	34.8 (8/23)	10 (1/10)	–
HLH	8.7 (2/23)	10 (1/10)	–
Unknown, number	3	3	–
Major treatment, %			
Corticosteroid	82.6 (19/23)	69.2 (9/13)	28.6 (4/14)
Anakinra	13.0 (3/23)	7.7 (1/13)	28.6 (4/14)
IVIg	17.4 (4/23)	7.7 (1/13)	7.1 (1/14)
NSAIDs	21.7 (5/23)	7.7 (1/13)	14.3 (2/14)
HCQ	4.3 (1/23)	15.4 (2/13)	7.1 (1/14)
No medication	–	–	57.1 (8/14)
Unknown, number	3	0	0
Delivery, %			
Transvaginal	66.7 (12/18)	50 (4/8)	62.5 (5/8)
Cesarean section	22.2 (4/18)	50 (4/8)	25 (2/8)
Abortion	11.1 (2/18)	0 (0/8)	12.5 (1/8)
Unknown, number	8	5	6
Major obstetrical and perinatal complications, %			
Preterm birth	32 (8/25)	33.3 (4/12)	16.7 (2/12)
IUGR	8 (2/25)	41.7 (5/12)	8.3 (1/12)
pPROM	12 (3/25)	8.3 (1/12)	8.3 (1/12)
No major complication	48 (12/25)	41.7 (5/12)	50 (6/12)
Others, number	2 for OHD, 1 for neonatal HLH, 1 for preeclampsia, and 1 for neonatal death for IRDS	1 for GDM, and 1 for OHD	1 for neonatal HLH, 1 for GDM, and 1 for HDP
Unknown, number	1	1	2

The median age of patients at pregnancy was almost 30 years (median [interquartile range (IQR)]: 29 [25–33], 31 [29–34], and 27 [26–30] for the first-onset, recurrent-flare, and no-flare types, respectively), indicating that women with AOSD during pregnancy were younger than the general population at first diagnosis [1]. Regarding parity, AOSD flare mainly happens among primigravida patients, with the recurrent-flare type obtaining the highest rate (83.3%), although all patients in this type are in clinical remission at conception [4, 7, 9, 13, 14, 16, 25, 31]. Our case was also in clinical remission and did not require any medication at conception.

Most likely, the AOSD flare occurred until second trimester and at postpartum, with limited reports of flare at third trimester; the first-onset type was found only in two cases [21, 24] and the recurrent-flare type in one case, which is our case. Although AOSD flare occurring at third trimester is extremely rare, the clinical symptoms of AOSD are similar to those of HELLP syndrome and AFLP, suggesting that close attention to perinatal management is crucial. Generally, the Yamaguchi criteria [2] is adopted for the diagnosing AOSD (Table 1). However, we needed to assess AOSD recurrence in a comprehensive way by considering the clinical features and laboratory findings because no criteria for the diagnosis of AOSD recurrence are currently available. In fact, the review showed that most of the major symptoms of AOSD detected in the recurrent-flare type are relatively fewer than in the first-onset type. In addition, the major symptoms and findings of AOSD are elevated liver enzyme, leukocytosis, and sometimes abdominal pain or nausea, which are difficult to distinguish from HELLP syndrome or AFLP, particularly in cases with flare occurring at third trimester. Elevated ferritin [2] and IL-18 [33], which are reportedly biomarkers of AOSD activity, are considered better diagnostic markers than any other markers. Specific AOSD features such as fever, splenomegaly, and typical rash can support the possibility of AOSD. Izuka et al. recently reported that elevated serum aldolase can be a useful biomarker of AOSD [34]. In our case, the serum aldolase level was high (30.6 U/L), which supported the diagnosis of recurrent AOSD flare. Finally, we and some rheumatologists comprehensively diagnosed the patient with AOSD recurrence flare in consideration of the AOSD findings such as elevated ferritin, IL-18, and aldolase levels, splenomegaly, and uneventful fetal status.

AOSD treatments generally include PSL/mPSL, nonsteroidal anti-inflammatory agents (NSAIDs) and in more severe cases, methotrexate, anakinra (interleukin-1 [IL-1] inhibitor), intravenous immune globulin (IVIg), cyclosporine, or tacrolimus. However, selecting the appropriate medication for pregnant patients entails some limitations. Jamilloux Y et al. recommended corticosteroids as the first treatment choice, and IVIg and IL-1 inhibitor as the second treatment choice for AOSD [35]. Our review also showed that corticosteroid was mainly used, followed by IL-1 inhibitor and IVIg. In Japan, where most of the IL-1 antagonists have not yet been approved, tacrolimus may be a reasonable second choice for severe AOSD flare in pregnancy, and some successful management cases reported to have used this medication during pregnancy [11, 23]. In our case, twice mPSL pulse therapy followed by oral PSL was chosen; consequently, pregnancy was maintained until 36 gestational weeks.

In almost one-third of cases in both the first-onset and recurrent-flare types, the main obstetrical complication was preterm birth. Some reports showed that other autoimmune diseases or autoinflammatory diseases, such as systemic lupus erythematosus and rheumatoid arthritis, occurring during pregnancy are risk factors for preterm birth, and maternal disease activity contributes to the increased risk of preterm delivery [36–38]. Furthermore, long-term steroidal use is another risk factor for preterm birth [37, 38]. Our review results showed that the incidence of preterm delivery in both the first-onset and recurrent-flare types of AOSD was almost twice higher than that in the no-flare type. Therefore, the high disease activity of AOSD and long-term use of systemic steroids may be possible reasons for the high incidence of preterm delivery in patients with AOSD. Our case also delivered prematurely, with high disease activity and long-term systemic steroid use including twice steroid pulse therapies. Hemophagocytic lymphohistiocytosis (HLH) is also a severe and crucial complication of AOSD. HLH was diagnosed in some cases having the first-onset and recurrent-flare types and in neonatal cases delivered from mothers with AOSD, even in clinical remission. Meanwhile, we believe that in general, the overall severity of AOSD during pregnancy may be better than the HELLP syndrome and AFLP. In fact, lethal complication was only found in one case wherein a premature fetus died at 28 weeks old because of infantile respiratory distress syndrome (IRDS) in the first-onset type, as reported by Green et al. in 1982 [8]. Therefore, with the cooperation of rheumatologists and pediatricians, pregnant patients with AOSD can maintain their pregnancy by paying special attention to prematurity, fetal growth, and HLH, even if the case is in clinical remission.

The relationship of pathogenesis between AOSD and pregnancy remains unclear. Ida A et al. reported that serum IL-18 levels were significantly elevated in pregnant women from the first trimester until the onset of labor than in nonpregnant women [39]. They noticed further increment of serum IL-18 levels after labor, lasting until at least the third day of postpartum. This result parallels the clinical feature of AOSD in pregnancy, that is, AOSD flares are likely to happen in the first/second trimester and postpartum. Considering that our case had also prominently high IL-18 levels, IL-18 elevation may cause the flare of AOSD during pregnancy, suggesting that pregnancy may be a risk factor of AOSD. Therefore, the serum IL-18 levels as a follow-up during pregnancy may be a useful predictive and diagnostic marker of AOSD flare; however, further investigations are required.

From the abovementioned consideration, patients with AOSD can be pregnant and continue pregnancy safely while under treatment, considering that the prognosis of pregnant women with AOSD and their fetuses is favorable in previous cases. Furthermore, for a safer pregnancy in patients complicated with AOSD, we, as obstetricians, need tight cooperation with rheumatologists and pediatricians for the sufficient control of AOSD activity and perinatal care and should provide enough explanation to patients on the risks of flares during pregnancy. Moreover, we conduct frequent laboratory tests and careful follow-up in preparation for flares, premature birth, and HLH. If the flare happens at third trimester, as in our case, we need to differentiate AOSD from HELLP syndrome and AFLP because of the similarity of laboratory data and physical findings. Fever, splenomegaly, elevated ferritin and IL-18 levels, and fetal status could be useful findings for the differentiation from HELLP syndrome and AFLP.

To our knowledge, this report is the first to present the recurrent flare of AOSD during pregnancy at third trimester. However, we need to further investigate on the proper management of pregnant women with AOSD.

## Data Availability

The datasets that support the findings about this case are available from the corresponding author, SH, on reasonable request.

## Abbreviations

AOSD: Adult-onset Still’s disease
TNF-α: Tumor necrosis factor-α
IL-6: Interleukin-6
IL-18: Interleukin-18
HELLP: Hemolysis, elevated liver enzymes and low platelet count
AFLP: Acute fatty liver of pregnancy
GDM: Gestational diabetes mellitus
EFBW: Estimated fetal body weight
SD: Standard deviation
mPSL: Methylprednisolone
PSL: Prednisolone
IUGR: Intrauterine growth restriction
pPROM: Preterm premature rupture of membrane
CRP: C-reactive protein
IQR: Interquartile range
NSAIDs: Nonsteroidal anti-inflammatory agents
IL-1: Interleukin-1
IVIg: Intravenous immune globulin
HLH: Hemophagocytic lymphohistiocytosis
IRDS: Infantile respiratory distress syndrome
NVD: Normal vaginal delivery
T-Bil: Total bilirubin
AST: Aspartate aminotransferase
ALT: Alanine aminotransferase
LDH: Lactate dehydrogenase
γ-GTP: γ-glutamyl transpeptidase
BUN: Blood urea nitrogen
Cre: Creatinine
BS: Blood sugar
TG: Triglyceride
WBC: White blood cell
RBC: Red blood cell
Hb: Hemoglobin
Ht: Hematocrit
Plt: Platelet
PT-INR: Prothrombin-time international normalized ratio
APTT: Activated partial thromboplastin time
Fib: Fibrinogen
AT III: Antithrombin III
HCQ: Hydroxychloroquine
OHD: Oligohydramnios
HDP: Hypertensive disorders of pregnancy

## References

[1] Gerfaud-Valentin M, Jamilloux Y, Iwaz J, Sève P (2014). Adult-onset Still’s disease. Autoimmun Rev.

[2] Yamaguchi M, Ohta A, Tsunematsu T, Kasukawa R, Mizushima Y, Kashiwagi H (1992). Preliminary criteria for classification of adult Still’s disease. J Rheumatol.

[3] Plaçais L, Mekinian A, Bornes M, Poujol-Robert A, Bigé N, Maury E, Fain O (2018). Adult onset Still’s disease occurring during pregnancy: case-report and literature review. Semin Arthritis Rheum.

[4] Gerfaud-Valentin M, Hot A, Huissoud C, Durieu I, Broussolle C, Seve P (2014). Adult-onset Still’s disease and pregnancy: about ten cases and review of the literature. Rheumatol Int.

[5] Sibai BM (2004). Diagnosis, controversies, and management of the syndrome of hemolysis, elevated liver enzymes, and low platelet count. Obstet Gynecol.

[6] Ch’ng CL, Morgan M, Hainsworth I, Kingham JG (2002). Prospective study of liver dysfunction in pregnancy in Southwest Wales. Gut..

[7] Stein GH, Cantor B, Panush RS (1980). Adult Still’s disease associated with pregnancy. Arthritis Rheum.

[8] Green J, Kanter Y, Barzilai D (1982). Adult Still’s disease associated with pregnancy. Isr J Med Sci.

[9] Mok MY, Lo Y, Leung PY, Lau CS (2004). Pregnancy outcome in patients with adult onset Still’s disease. J Rheumatol.

[10] Smith CJF, Chambers CD (2018). Five successful pregnancies with antenatal anakinra exposure. Rheumatol Oxf Engl.

[11] Nakamura H, Odani T, Shimizu Y, Takeda T, Kikuchi H (2016). Usefulness of tacrolimus for refractory adult-onset Still’s disease: report of six cases. Mod Rheumatol.

[12] Moussa M, Hassan MF (2014). Newly diagnosed adult-onset Still’s disease with pure red cell aplasia in pregnancy. Arch Gynecol Obstet.

[13] Shimizu M, Sakakibara Y, Kawano M, Yachie A (2012). Transient impairment of NK cell function in an infant born to a mother with adult-onset Still’s disease: perinatal effect of maternal IL-18. Clin Immunol.

[14] Le Loët X, Daragon A, Duval C, Thomine E, Lauret P, Humbert G (1993). Adult onset Still’s disease and pregnancy. J Rheumatol.

[15] Parry G, Goudevenos J, Williams DO (1992). Coronary thrombosis postpartum in a young woman with Still’s disease. Clin Cardiol.

[16] Berger CT, Recher M, Steiner U, Hauser TM (2009). A patient’s wish: anakinra in pregnancy. Ann Rheum Dis.

[17] Fischer-Betz R, Specker C, Schneider M (2011). Successful outcome of two pregnancies in patients with adult-onset Still’s disease treated with IL-1 receptor antagonist (anakinra). Clin Exp Rheumatol.

[18] Kaplinsky N, Pras M, Frankl O (1980). An adult form of juvenile rheumatoid arthritis. Arch Intern Med.

[19] Yebra Bango M, García Paez JM, Solovera JJ, Merino MF, Girón González JA (1985). Adult-onset Still’s disease: a case with onset during pregnancy. Arthritis Rheum.

[20] Falkenbach A, Lembcke B, Schneider M, Wigand R, Mulert-Ernst R, Caspary W (1994). Polyserositis in adult Still’s disease with onset during pregnancy [corrected]. Clin Rheumatol.

[21] Mahmud T, Hughes GR (1999). Intravenous immunoglobulin in the treatment of refractory adult Still’s disease. J Rheumatol.

[22] Liozon E, Ly K, Aubard Y, Vidal E (1999). Intravenous immunoglobulins for adult Still’s disease and pregnancy. Rheumatol Oxf Engl..

[23] Yamamoto M, Tabeya T, Suzuki C, Naishiro Y, Yajima H, Shimizu Y (2012). Adult-onset Still’s disease in pregnancy. Mod Rheumatol.

[24] Lin A, Ma TP, Cheng FW, Ng PC (2016). Neonatal haemophagocytic lymphohistiocytosis associated with maternal adult-onset Still’s disease. Neonatology..

[25] De Miguel E, Cuesta M, Martín-Mola E, Gijòn-Baños J (1992). Adult Still’s disease and pregnancy. J Rheumatol.

[26] Sayarlioglu M, Sahin M, Cetin GY, Avan R, Cerit M (2010). Maternal exposure to leflunomide and methotrexate in a patient with adult-onset Still’s disease. Rheumatol Oxf Engl..

[27] Park JH, Kim SH, Kim HJ, Lee SJ, Jeong DC, Kim SY (2015). Macrophage activation syn- drome in a newborn infant born to a mother with autoimmune disease. J Perinatol.

[28] Pan VL, Haruyama AZ, Guberman C, Kitridou RC, Wing DA (2003). Newly diagnosed adult onset still disease in pregnancy. Obstet Gynecol.

[29] Odai T, Isozaki T, Kasama T, Ogata H, Kinugasa E (2015). Therapeutic efficacy of leukocytapheresis in a pregnant woman with refractory adult-onset Still’s disease. Intern Med.

[30] Katz WE, Starz TW, Winkelstein A (1990). Recurrence of adult Still’s disease after pregnancy. J Rheumatol.

[31] Dunn T, Cho M, Medeiros B, Logan A, Ungewickell A, Liedtke M (2012). Hemophagocytic lymphohistiocytosis in pregnancy: a case report and review of treatment options. Hematology..

[32] De Carolis S, Cianci F, Del Sordo G, Garofalo S, Garufi C, Lanzone A (2019). Adult onset Still’s disease and pregnancy. Autoimmun Rev.

[33] Colafrancesco S, Priori R, Alessandri C, Perricone C, Pendolino M, Picarelli G, Valesini G (2012). IL-18 serum level in adult onset Still’s disease: a marker of disease activity. Int J Inflam.

[34] Izuka S, Yamashita H, Takahashi Y, Kaneko H. Serum aldolase serves as a useful marker for diagnosis and assessment of disease activity in patients with adult-onset Still’s disease. Clin Exp Rheumatol. 2020;38 Suppl 127(5):119. 32105595

[35] Jamilloux Y, Gerfaud-Valentin M, Henry T, Sève P (2015). Treatment of adult-onset Still’s disease: a review. Ther Clin Risk Manag.

[36] Buyon JP, Kim MY, Guerra MM, Laskin CA, Petri M, Lockshin MD (2015). Predictors of pregnancy outcomes in patients with lupus: a cohort study. Ann Intern Med.

[37] Smith CJF, Förger F, Bandoli G, Chambers CD (2019). Factors associated with preterm delivery among women with rheumatoid arthritis and women with juvenile idiopathic arthritis. Arthritis Care Res.

[38] Clark CA, Spitzer KA, Nadler JN, Laskin CA (2003). Preterm deliveries in women with systemic lupus erythematosus. J Rheumatol.

[39] Ida A, Tsuji Y, Muranaka J, Kanazawa R, Nakata Y, Adachi S (2000). IL-18 in pregnancy; the elevation of IL-18 in maternal peripheral blood during labour and complicated pregnancies. J Reprod Immunol.

